# Psychometric evaluation of the Chinese CNAAQ-2: factor structure and measurement invariance across gender and educational stages

**DOI:** 10.3389/fpsyg.2026.1882376

**Published:** 2026-09-09

**Authors:** Wei He, Shuaiqi Jia, Jinying Wu, Minyan Duan, Xiancheng Zeng

**Affiliations:** Guangzhou Sport University, Guangzhou, China

**Keywords:** athletic ability beliefs, Chinese youth, CNAAQ-2-C, measurement, psychometric validation

## Abstract

Beliefs about the nature of athletic ability may shape motivation and engagement in sport, yet their measurement has received limited examination in Chinese populations. This research adapted and evaluated the Chinese version of the Conceptions of the Nature of Athletic Ability Questionnaire-2 (CNAAQ-2-C) across three studies. In Study 1, ordinal exploratory factor analysis with 592 university students supported four factors, Stable, Gift, Improvement, and Learning, χ^2^(24) = 90.76, CFI = 0.987, TLI = 0.964, RMSEA = 0.069, and SRMR = 0.019. Study 2 replicated this structure in an independent sample of 282 university students; the correlated four-factor model showed good fit, χ^2^(48) = 84.89, CFI = 0.979, TLI = 0.971, RMSEA = 0.052, and SRMR = 0.040, with standardized loadings from 0.645 to 0.932. Study 3 examined measurement invariance using the complete 12-item model. Configural, loading, and threshold invariance were supported across gender. Across university, primary school, and junior high school samples, configural and loading invariance were supported, whereas evidence for full threshold invariance was mixed. Overall, the CNAAQ-2-C provides a useful four-factor measure for group-level research, although mean comparisons across educational stages require caution.

## 1 Introduction

Beliefs about the nature of athletic ability provide a framework through which young people interpret success, failure, and improvement in sport and physical activity. These beliefs are commonly distinguished as entity beliefs, which view athletic ability as relatively fixed, and incremental beliefs, which view it as developable through effort, learning, and practice (Biddle et al., 2003; Dweck, 2013). This distinction is grounded in the broader implicit theories framework, according to which beliefs about ability are associated with how individuals set goals, respond to setbacks, and regulate their motivation in achievement settings (Burnette et al., 2013). Athletic ability beliefs are particularly relevant in youth sport, where children and adolescents repeatedly encounter evaluation, social comparison, setbacks, and opportunities for long-term skill development (Jang et al., 2024). Entity beliefs have generally been associated with less adaptive motivational patterns, including avoidance of difficult tasks, lower enjoyment, and weaker persistence (Danthony et al., 2020; Lyons et al., 2015; Warburton et al., 2021). By contrast, incremental beliefs have more often been linked to mastery-oriented goals, self-efficacy, willingness to undertake challenging tasks, and continued engagement in sport and physical activity (Mascret et al., 2016; Ommundsen, 2006; Orvidas et al., 2018; Wang et al., 2005). More recent evidence further supports the domain-specific relevance of athletic ability beliefs to young people’s responses to failure, skill development, and participation in physical activity (Greule et al., 2024; Jia et al., 2024; Kyler and Moscicki, 2024). Together, these findings highlight the value of a psychometrically sound measure of athletic ability beliefs for youth populations.

The Conceptions of the Nature of Athletic Ability Questionnaire-2 (CNAAQ-2) (Biddle et al., 2003) is a widely used measure of beliefs about athletic ability in sport and physical activity contexts. The instrument contains 12 items representing four first-order dimensions. Stable beliefs refer to the view that athletic ability is relatively resistant to change, whereas Gift beliefs emphasise the importance of innate qualities for sporting competence. Improvement beliefs reflect the expectation that ability can develop through effort and practice, while Learning beliefs emphasise the acquisition of skills through learning and training. Stable and Gift are commonly grouped under the broader theoretical category of entity beliefs, whereas Improvement and Learning are grouped under incremental beliefs. The CNAAQ-2 has subsequently been used in a range of sport and physical activity studies and has generally shown support for its multidimensional structure and internal consistency (Danthony et al., 2020; Gardner et al., 2017; McNeil et al., 2024). Nevertheless, evidence obtained in previous samples cannot establish that the same structure will be retained following translation or when the measure is administered in a different cultural and educational context.

The CNAAQ-2 has been used in several linguistic and cultural settings, including samples from Singapore, the Netherlands, and South Korea (Jang et al., 2024; Wang et al., 2005; Weltevreden et al., 2022). These studies provide evidence that the general framework of athletic ability beliefs can be examined across different populations, but they also indicate that the psychometric performance of particular items and dimensions may vary across contexts. Cross-cultural adaptation therefore involves more than producing a linguistically accurate translation. It also requires consideration of conceptual equivalence—whether respondents in the target population interpret the constructs and item content in ways that are sufficiently consistent with the intended meanings of the original instrument (Putnick and Bornstein, 2016). This issue is especially relevant to the CNAAQ-2 because terms such as ability, gift, effort, improvement, and learning may carry different connotations across linguistic, cultural, and educational contexts. Accordingly, evidence from previous versions cannot be assumed to establish the validity of a Mandarin Chinese adaptation, and the factor structure and measurement properties of the CNAAQ-2-C require direct evaluation in Chinese samples.

A further question is whether the CNAAQ-2-C measures athletic ability beliefs comparably across gender and educational-stage groups. Meaningful group comparisons require evidence that the same latent constructs are represented by a similar factor structure and comparable item–factor relations; otherwise, observed differences may partly reflect measurement non-invariance rather than substantive differences between groups (Putnick and Bornstein, 2016; Vandenberg and Lance, 2000). Comparability across educational stages cannot be assumed, because students may differ in their comprehension of questionnaire wording, interpretation of abstract ability concepts, accumulated sport experience, and use of response categories (Borgers et al., 2000). Gender comparability likewise requires direct examination within each educational-stage group rather than evidence from a single sample. Measurement invariance testing is therefore needed to determine the extent to which the CNAAQ-2-C can support comparisons of latent means and structural relations across gender and educational-stage groups.

### 1.1 The present study

The present study had two related aims. The first was to evaluate the factor structure and internal consistency of the Chinese version of the CNAAQ-2 (CNAAQ-2-C). The second was to examine whether the scale operates comparably across gender and educational-stage groups. Three studies were conducted. Study 1 used ordinal exploratory factor analysis to investigate the dimensional structure of the CNAAQ-2-C in a university student sample. Study 2 used confirmatory factor analysis with ordered categorical indicators in an independently recruited university sample to evaluate the four-factor structure identified in Study 1 and compare it with the theoretically proposed higher-order structure. Study 3 examined gender measurement invariance separately within the university, primary school, and junior high school samples, as well as measurement invariance across the three educational-stage groups, using the complete 12-item correlated four-factor model. Together, the three studies provided an initial, staged evaluation of the factor structure, internal consistency, and cross-group measurement comparability of the CNAAQ-2-C in Chinese student samples.

## 2 Method

### 2.1 Participants

The present research included three studies involving Chinese youth from different educational-stage groups. Study 1 recruited 687 university students from six universities, between September and October 2023. After excluding invalid responses (e.g., straight-lining and alternating response patterns; Lelkes et al., 2012), the final sample consisted of 592 students (*M*age = 19.70, *SD* = 1.25, range = 17–27), including 362 men. Study 2 recruited 300 university students from a university in March 2024. After excluding 18 invalid responses, the final sample consisted of 282 students (*M*age = 20.09, *SD* = 0.94, range = 18–23), including 215 men.

Study 3 recruited 634 students from two primary schools and one junior high school between April 2024 and June 2025. After excluding invalid responses, the final sample comprised 538 students (*M*age = 12.02, *SD* = 0.97, range = 10–14), including 252 boys. The sample included 305 primary school students (*M*age = 11.37, *SD* = 0.73; 148 boys) and 233 junior high school students (*M*age = 12.88, SD = 0.43; 104 boys). Educational-stage measurement invariance was examined using these participants and the 592 university students from Study 1, yielding a total analytic sample of 1130 participants.

The protocol for all three studies was approved by the Ethics Committee of Guangzhou Sport University (Approval No. 2023LCLL-65). University students provided informed consent before participation. For participants who were minors, written informed consent was obtained from their parents or legal guardians, and student assent and school permission were obtained before data collection. Participation was voluntary. Questionnaires were completed anonymously, and the data were securely stored and accessible only to the research team.

*Data screening*. Questionnaires were excluded when responses were incomplete to the extent that the relevant scale scores could not be calculated or when the response pattern indicated insufficient engagement with the questionnaire. Patterned responding included evident straight-lining and mechanically repetitive response sequences. The same screening principles were applied across the three studies.

### 2.2 Measures

#### 2.2.1 The Chinese version of the CNAAQ-2

The Chinese version of the Conceptions of the Nature of Athletic Ability Questionnaire-2 (CNAAQ-2-C) was developed and validated in the present research. The original CNAAQ-2 consists of 12 items assessing two higher-order dimensions, entity beliefs and incremental beliefs, and four first-order factors: Stable, Gift, Improvement, and Learning. Items are rated on a 5-point Likert scale ranging from 1 (strongly disagree) to 5 (strongly agree).

### 2.3 Procedure

The CNAAQ-2 was translated into Chinese using a multistep forward- and back-translation procedure drawing on established cross-cultural adaptation guidelines (Brislin, 1980; Beaton et al., 2000). Four Chinese-English bilingual researchers independently translated the original English items into Chinese. The translators had backgrounds in sport psychology, education, and English language studies. The four translations were compared item by item and reconciled through discussion, with attention to conceptual equivalence, linguistic clarity, and appropriateness for Chinese respondents, and differences in wording were resolved through discussion and consensus.

Two additional bilingual researchers, who had not participated in the forward translation, independently back-translated the reconciled Chinese version into English. The back-translations were compared with the original items, and remaining wording differences were resolved through consensus. The complete English and Chinese item versions are provided in the Supplementary material (see Supplementary Table S1).

Study 1 was conducted from September to October 2023, and Study 2 was conducted in March 2024. In both studies, university students completed an online questionnaire after providing informed consent. Study 3 was conducted from April 2024 to June 2025. Primary and junior high school students completed a paper-and-pencil questionnaire once in their classrooms. Written parental consent and student assent were obtained, and permission was granted by the participating schools. For the educational-stage measurement-invariance analysis, the school-age sample from Study 3 was analysed together with the university sample from Study 1.

### 2.4 Data analysis

Descriptive statistics and Cronbach’s alpha (α) were computed in SPSS 25.0. McDonald’s omega (ω), exploratory factor analysis (EFA), confirmatory factor analysis (CFA), and measurement invariance analyses were conducted in Mplus 8.3. Because the CNAAQ-2-C items used five-point response scales, they were treated as ordered categorical indicators and estimated using weighted least squares mean- and variance-adjusted estimation (WLSMV).

In Study 1, EFA with Geomin oblique rotation was used to compare one- to four-factor solutions. In Study 2, CFA was conducted in an independent sample to test the 12-item correlated four-factor model. Model fit was evaluated using the chi-square statistic (χ^2^), comparative fit index (CFI), Tucker–Lewis index (TLI), root mean square error of approximation (RMSEA), and standardized root mean square residual (SRMR), with the indices considered jointly (Hu and Bentler, 1999).

In Study 3, multigroup CFA based on the complete 12-item four-factor model tested configural, loading, and threshold invariance across gender and educational-stage groups. Measurement invariance for the ordered categorical items was evaluated within a WLSMV multigroup CFA framework, following recommendations for the identification and testing of invariance models with categorical outcomes (Svetina et al., 2020; Wu and Estabrook, 2016). Changes in CFI, RMSEA, and SRMR were considered jointly. A decrease in CFI of no more than 0.010, an increase in RMSEA of no more than 0.015, and increases in SRMR of no more than 0.030 for loading invariance and 0.010 for threshold invariance were used as conventional guidelines (Chen, 2007; Cheung and Rensvold, 2002). These criteria were treated as approximate rather than absolute, given evidence that the performance of changes in fit indices under WLSMV depends on model and sample characteristics (Sass et al., 2014).

## 3 Results

### 3.1 Study 1: exploratory factor analysis of the CNAAQ-2-C

An exploratory factor analysis with Geomin oblique rotation was conducted on the 12 CNAAQ-2-C items using WLSMV estimation. One- through four-factor solutions were examined. As shown in Table 1, model fit improved as additional factors were extracted, and the four-factor solution provided the best fit, χ^2^(24) = 90.76, *p* < 0.001, CFI = 0.987, TLI = 0.964, RMSEA = 0.069, and SRMR = 0.019. In comparison, the one- and two-factor solutions showed poor fit, while the three-factor solution remained inadequate in terms of RMSEA and TLI. The four-factor solution was therefore retained for interpretation.

**Table 1:** Results of different factors model of EFA (Study 1).

Model	χ^2^	*df*	RMSEA	CFI	TLI	SRMR
One factor	2433.172	54	0.273	0.529	0.424	0.208
Two factors	769.473	43	0.169	0.856	0.779	0.078
Three factors	281.579	33	0.113	0.951	0.902	0.041
Four factors	90.756	24	0.069	0.987	0.964	0.019

The rotated loading pattern was broadly consistent with the proposed Stable, Gift, Improvement, and Learning dimensions (Table 2). Primary loadings ranged from 0.587 to 0.841 for Stable, from 0.397 to 0.887 for Gift, from 0.749 to 0.820 for Improvement, and from 0.764 to 0.797 for Learning. Most cross-loadings were small. Item 4 had a comparatively modest loading of 0.397 on Gift and a cross-loading of 0.306 on Learning, indicating a less clearly differentiated factor association than the other items. Internal consistency ranged from 0.74 to 0.81 for Cronbach’s α and from 0.75 to 0.81 for McDonald’s ω. Overall, the findings provided preliminary support for the four-factor structure, which was subsequently examined in an independent sample in Study 2.

**Table 2:** The results of descriptive statistics, internal consistencies, Geomin rotated loadings in EFA (Study 1).

Item	*M*	*SD*	Ske	Kur	Response Frequency					Reliability		Geomin Rotated Loading			
					1	2	3	4	5	𝛼	ω	Factor 1	Factor 2	Factor 3	Factor 4
Stable	3.060	0.944	0.038	−0.376						0.75	0.75				
Item 1	3.010	1.148	0.024	−0.597	67	113	233	106	73			**0.668**	0.005	−0.051	0.006
Item 3	2.980	1.163	0.13	−0.658	65	134	221	94	78			**0.841**	0.017	0.026	−0.001
Item 10	3.190	1.154	−0.053	−0.751	45	117	203	132	95			**0.587**	0.231	0.042	−0.006
Gift	3.503	0.884	−0.151	−0.212						0.74	0.75				
Item 4	3.530	1.146	−0.362	−0.649	31	76	179	161	145			0.231	**0.397**	−0.120	0.306
Item 7	3.540	1.049	−0.297	−0.406	22	59	214	170	127			0.062	**0.626**	0.060	0.072
Item 11	3.440	1.063	−0.171	−0.54	23	78	222	155	114			−0.011	**0.887**	−0.001	−0.028
Improvement	3.372	0.921	−0.225	−0.059						0.81	0.81				
Item 6	3.270	1.096	−0.133	−0.507	38	89	231	141	93			−0.038	0.046	**0.749**	−0.034
Item 9	3.410	1.086	−0.202	−0.584	27	86	208	161	110			0.006	−0.004	**0.820**	0.088
Item 12	3.440	1.071	−0.281	−0.36	31	63	225	163	110			0.025	−0.026	**0.799**	0.012
Learning	4.064	0.843	−0.726	0.042						0.78	0.78				
Item 2	4.100	1.052	−0.981	0.153	13	36	113	144	286			0.011	−0.081	−0.053	**0.797**
Item 5	4.000	1.011	−0.776	−0.053	11	34	133	178	236			0.075	−0.002	0.073	**0.764**
Item 8	4.080	0.975	−0.906	0.392	12	19	129	179	253			−0.180	0.187	0.046	**0.780**

Ske = Skewness, Kur = Kurtosis. 1 = strongly disagree, 2 = disagree, 3 = neither agree nor disagree, 4 = agree, 5 = strongly agree.

### 3.2 Study 2: confirmatory factor analysis and sensitivity analyses

Descriptive statistics, correlations, and internal consistency estimates are presented in Tables 3, 4. Internal consistency coefficients ranged from 0.700 to 0.775 for Cronbach’s α and from 0.709 to 0.775 for McDonald’s ω. Learning showed a relatively high mean (*M* = 4.36, *SD* = 0.71) and a negatively skewed distribution, indicating greater endorsement of learning-oriented beliefs.

**Table 3:** The results of descriptive statistics of CNAAQ-2-C in CFA (Study 2).

Item	*M*	*SD*	Ske	Kur	Response Frequency				
					1	2	3	4	5
Stable									
Item 1	2.780	1.027	0.134	−0.225	33	70	122	40	17
Item 3	2.624	1.051	0.225	−0.422	44	83	103	39	13
Item 10	2.823	1.021	0.099	−0.415	28	77	109	53	15
Gift									
Item 4	3.564	1.039	−0.334	−0.343	10	27	99	86	60
Item 7	3.486	1.037	−0.116	−0.562	8	34	111	71	58
Item 11	3.521	1.048	−0.272	−0.439	10	31	101	82	58
Improvement									
Item 6	3.291	1.084	−0.042	−0.573	14	47	111	63	47
Item 9	3.284	1.102	−0.147	−0.470	19	39	113	65	46
Item 12	3.291	1.077	−0.119	−0.402	17	38	118	64	45
Learning									
Item 2	4.429	0.871	−1.767	3.131	4	9	21	76	172
Item 5	4.213	0.919	−1.017	0.397	2	13	44	87	136
Item 8	4.436	0.776	−1.348	1.557	1	5	29	82	165

Ske, Skewness; Kur, Kurtosis. 1 = strongly disagree, 2 = disagree, 3 = neither agree nor disagree, 4 = agree, 5 = strongly agree.

**Table 4:** Descriptive statistics of results (Study 2).

	Variables	*M*	*SD*	Ske	Kur	𝛼	ω	1	2	3	4	5
1	Stable	2.742	0.821	0.018	−0.198	0.708	0.710					
2	Gift	3.524	0.824	−0.063	−0.238	0.700	0.709	0.309^***^				
3	Improvement	3.288	0.903	−0.108	−0.06	0.775	0.775	−0.026	0.115			
4	Learning	4.359	0.706	−1.335	2.219	0.762	0.773	−0.048	0.302^***^	0.122^*^		
5	Gender	/	/	/	/	/	/	0.111	−0.001	0.040	−0.088	
6	Age	20.090	0.936	/	/	/	/	0.027	0.043	0.055	−0.011	−0.185^**^

^*^p < 0.05, ^**^p < 0.01, ^***^p < 0.001. Ske, Skewness, Kur, Kurtosis.

**Figure 1:**
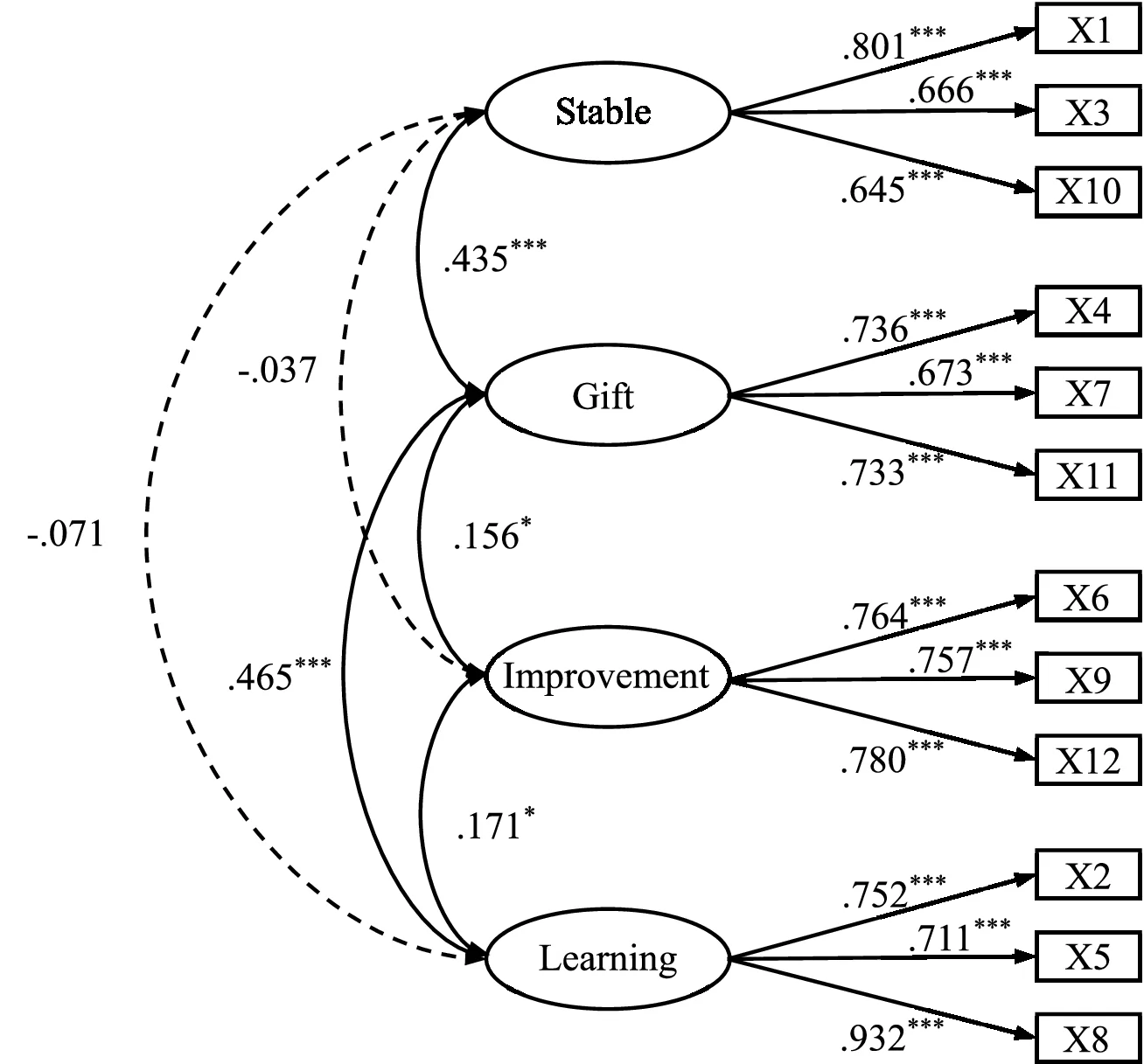
Result of confirmatory factor analysis among university students (Study 2). *p < 0.05, ***p < 0.001.

The 12-item correlated four-factor model, comprising Stable, Gift, Improvement, and Learning, was tested using WLSMV estimation. The model showed good fit, χ^2^(48) = 84.89, *p* < 0.001, CFI = 0.979, TLI = 0.971, RMSEA = 0.052, 90% CI [0.033, 0.070], and SRMR = 0.040. All items loaded significantly on their intended factors, with standardized factor loadings ranging from 0.645 to 0.932 (Figure 1). Notably, Item 4 had a standardized factor loading of 0.736 on Gift. The latent factor correlations ranged from −0.071 to 0.465, indicating that the four dimensions were related but empirically distinguishable.

A theoretically specified higher-order model was also examined, in which Stable and Gift were represented by Entity beliefs and Improvement and Learning by Incremental beliefs. Because each higher-order factor had only two first-order indicators, the corresponding unstandardized second-order loadings were constrained to equality for identification. This model showed substantially weaker fit, χ^2^(51) = 171.54, CFI = 0.931, TLI = 0.910, RMSEA = 0.092, 90% CI [0.077, 0.107], and SRMR = 0.069. Moreover, the standardized loadings of Improvement and Learning on Incremental were relatively modest (0.391 and 0.438, respectively). The correlated four-factor model was therefore retained as the preferred representation of the CNAAQ-2-C.

To further examine the performance of Item 4, two sensitivity models were estimated (see Table 5). First, an 11-item model excluding Item 4 was tested. Because Gift was represented by only two remaining indicators, their unstandardized loadings were constrained to equality for model identification. The 11-item model showed good fit, χ^2^(39) = 56.29, *p* = 0.036, CFI = 0.989, TLI = 0.985, RMSEA = 0.040, 90% CI [0.011, 0.061], and SRMR = 0.036. The Gift-Learning correlation decreased from 0.465 in the original model to 0.357 in the 11-item model.

**Table 5:** Model fit indices for the confirmatory factor analysis and item 4 sensitivity models (Study 2).

Model	χ^2^	*df*	RMSEA	CFI	TLI	SRMR
Competing measurement models						
Original 12-item correlated four-factor model	84.891	48	0.052	0.979	0.971	0.040
Two-higher-order-factor model^a^	171.538	51	0.092	0.931	0.910	0.069
Item 4 sensitivity models						
11-item model excluding Item 4^b^	56.288	39	0.040	0.989	0.985	0.036
12-item model with equal Gift-item loadings^c^	85.776	49	0.052	0.979	0.972	0.041

All models were estimated using WLSMV with the 12 items specified as ordered categorical indicators.

a In the higher-order model, Stable and Gift loaded on Entity, whereas Improvement and Learning loaded on Incremental. Because each higher-order factor had only two first-order indicators, the unstandardized second-order loadings within each higher-order factor were constrained to equality for identification.

b In the 11-item model, the unstandardized loadings of the two remaining Gift indicators were constrained to equality for identification.

c The same equality constraint on the two remaining Gift-item loadings was imposed in the 12-item auxiliary model. Because the 11- and 12-item models contained different sets of observed indicators, their fit indices were compared descriptively rather than through a formal chi-square difference test.

Because the 11-item model included both the deletion of Item 4 and an equality constraint, an additional 12-item model was estimated with the same equality constraint imposed on the other two Gift indicators. This model showed virtually identical fit to the original 12-item model, χ^2^(49) = 85.78, *p* < 0.001, CFI = 0.979, TLI = 0.972, RMSEA = 0.052, 90% CI [0.033, 0.069], and SRMR = 0.041. Thus, the descriptively better fit of the 11-item model was unlikely to be attributable solely to the equality constraint and was consistent with some overlap introduced by Item 4. Nevertheless, the original 12-item model already showed good fit, Item 4 loaded strongly on Gift in the independent CFA sample, and its removal would leave Gift with only two indicators and reduce the content coverage of the original scale. Item 4 was therefore retained, while its partial overlap with Learning is acknowledged.

### 3.3 Study 3: measurement invariance for CNAAQ-2-C

#### 3.3.1 Descriptive statistics and internal consistency

Descriptive statistics, correlations, and internal consistency estimates for the three educational-stage groups are presented in Table 6. Internal consistency was generally adequate in the university sample, with Cronbach’s α ranging from 0.744 to 0.807 and McDonald’s ω ranging from 0.749 to 0.809. Estimates were less consistent in the younger samples. In the primary school sample, α ranged from 0.691 to 0.750 and ω ranged from 0.701 to 0.751. In the junior high school sample, α ranged from 0.652 to 0.753 and ω ranged from 0.654 to 0.761, with the lowest estimates observed for Improvement. These results indicate greater measurement error for some three-item subscales in the younger groups.

**Table 6:** Descriptive statistics of results (Study 3).

	Variables	*M*	*SD*	Ske	Kur	𝛼	ω	1	2	3	4	5
University student (*n* = 592)												
1	Stable	3.060	0.944	0.038	−0.376	0.751	0.753					
2	Gift	3.503	0.884	−0.151	−0.212	0.744	0.749	0.514^***^				
3	Improvement	3.372	0.921	−0.225	−0.059	0.807	0.809	−0.078	0.120^***^			
4	Learning	4.064	0.843	−0.726	0.042	0.778	0.782	0.183^***^	0.431^***^	0.367^***^		
5	Gender	/	/	/	/	/	/	0.086^*^	−0.078	−0.148^***^	−0.091^*^	
6	Age	19.700	1.250	/	/	/	/	0.020	0.087^*^	0.185^***^	0.082^*^	−0.237^***^
Primary school student (*n* = 305)												
1	Stable	2.508	0.889	0.315	−0.230	0.730	0.730					
2	Gift	3.034	0.860	−0.017	−0.159	0.705	0.706	0.378^***^				
3	Improvement	3.935	0.790	−0.881	1.478	0.750	0.751	−0.356^***^	−0.220^***^			
4	Learning	4.054	0.683	−0.971	1.987	0.691	0.701	0.033	−0.040	0.439^***^		
5	Gender	/	/	/	/	/	/	0.023	−0.078	−0.168^**^	−0.088	
6	Age	11.365	0.732	/	/	/	/	−0.012	−0.023	−0.006	0.027	0.015
Junior high school student (*n* = 233)												
1	Stable	2.885	0.788	0.042	0.410	0.753	0.761					
2	Gift	3.245	0.772	0.121	−0.337	0.693	0.700	0.283^***^				
3	Improvement	3.433	0.673	0.173	0.234	0.652	0.654	−0.382^***^	−0.053			
4	Learning	3.905	0.614	−0.561	1.879	0.716	0.725	−0.040	0.182^**^	0.357^***^		
5	Gender	/	/	/	/	/	/	0.191^**^	0.008	−0.161^*^	−0.022	
6	Age	12.880	0.429	/	/	/	/	−0.050	−0.063	0.031	−0.003	0.030

^*^p < 0.05, ^**^p < 0.01, ^***^p < 0.001. Ske = Skewness, Kur = Kurtosis.

#### 3.3.2 Confirmatory factor analyses across educational-stage groups

**Figure 2:**
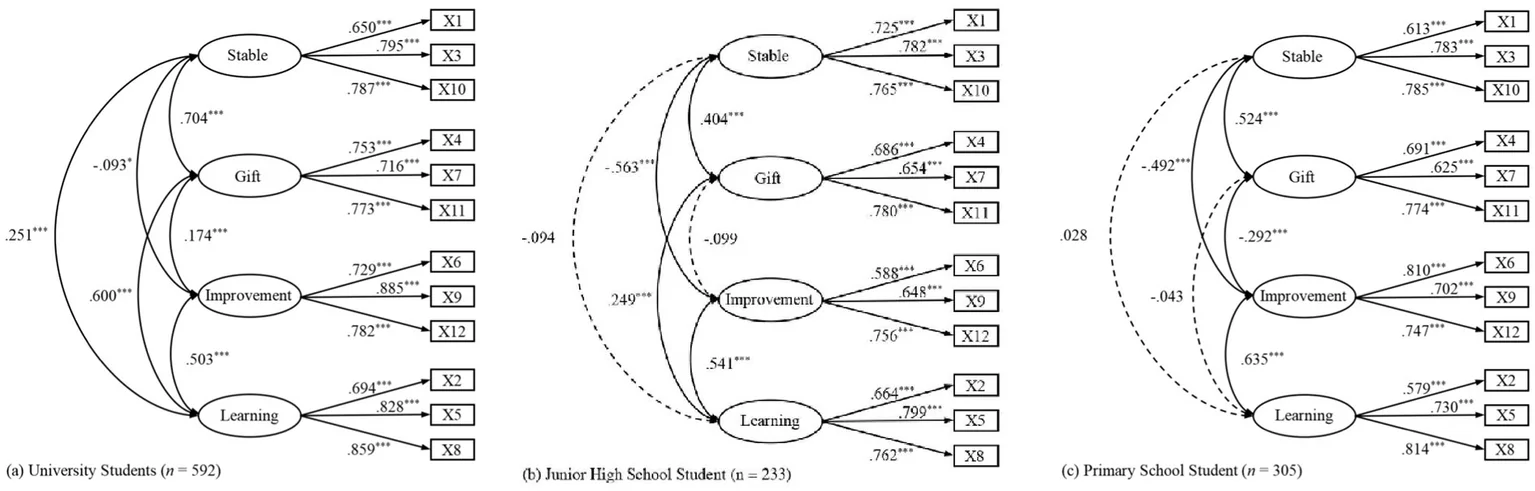
Group-specific confirmatory factor analysis models of the CNAAQ-2-C across educational-stage groups. **(a)** University students; **(b)** junior high school; **(c)** primary school students. Standardized factor loadings and factor correlations are shown. *p < 0.05, ***p < 0.001.

Before testing measurement invariance, the 12-item correlated four-factor model was examined separately in each educational-stage group using WLSMV estimation (Gregorich, 2006). The model showed good fit in the university sample, χ^2^(48) = 170.79, *p* < 0.001, CFI = 0.976, TLI = 0.967, RMSEA = 0.066, 90% CI [0.055, 0.077], and SRMR = 0.034. Standardized factor loadings ranged from 0.650 to 0.885 (see Figure 2a).

The model also showed good fit in the primary school sample, χ^2^(48) = 88.88, *p* < 0.001, CFI = 0.974, TLI = 0.964, RMSEA = 0.053, 90% CI [0.035, 0.070], and SRMR = 0.038. Standardized factor loadings ranged from 0.579 to 0.814 (see Figure 2c).

Fit was comparatively weaker in the junior high school sample, χ^2^(48) = 119.32, *p* < 0.001, CFI = 0.949, TLI = 0.930, RMSEA = 0.080, 90% CI [0.062, 0.098], and SRMR = 0.047 (see Figure 2b). Nevertheless, all items loaded significantly on their intended factors, with standardized loadings ranging from 0.588 to 0.799. Thus, the four-factor structure was interpretable in all three groups, although its performance was less optimal in the junior high school sample (Table 7).

**Table 7:** Measurement invariance of CNAAQ-2-C (Study 3).

Invariance	χ^2^	*df*	RMSEA	△RMSEA	CFI	△CFI	SRMR	ΔSRMR
Across gender (University Students, *n _Male_* = 362, *n _Female_ =* 230)								
Configural Invariance	269.359	96	0.078	/	0.969	/	0.042	/
Metric Invariance	279.046	104	0.075	−0.003	0.969	<0.001	0.043	0.001
Scalar Invariance	309.828	136	0.066	−0.009	0.969	<0.001	0.044	0.001
Across gender (Junior High School Students, *n _Male_* = 104, *n _Female_ =* 129)								
Configural Invariance	120.525	96	0.047	/	0.950	/	0.062	/
Metric Invariance	131.463	104	0.048	0.001	0.945	−0.005	0.077	0.015
Scalar Invariance	141.506	112	0.048	<0.001	0.940	−0.005	0.080	0.003
Across gender (Primary School Students, *n _Male_* = 148, *n _Female_ =* 157)								
Configural Invariance	124.635	96	0.044	/	0.962	/	0.054	/
Metric Invariance	129.988	104	0.040	−0.004	0.965	0.003	0.061	0.007
Scalar Invariance	137.276	112	0.038	−0.002	0.966	0.001	0.062	0.001
Across educational-stage groups (*n _Primary School Students_* = 305, *n _Junior High School Students_* = 233, *n _University Students_ =* 592)								
Configural invariance	373.061	144	0.065	/	0.973	/	0.038	/
Metric invariance	388.297	160	0.062	−0.003	0.973	<0.001	0.039	0.001
Scalar invariance	591.682	224	0.066	0.004	0.956	−0.017	0.043	0.004

ΔRMSEA, ΔCFI, and ΔSRMR represent changes relative to the preceding model. For the ordered categorical indicators, loading invariance imposed equality constraints on factor loadings, and threshold invariance additionally constrained item thresholds across groups.

#### 3.3.3 Gender measurement invariance within educational-stage groups

Gender measurement invariance was examined using the complete university sample of 362 men and 230 women. The configural model showed acceptable fit, χ^2^(96) = 269.36, *p* < 0.001, CFI = 0.969, RMSEA = 0.078, and SRMR = 0.042. Constraining the factor loadings to equality did not meaningfully reduce model fit, ΔCFI < 0.001, ΔRMSEA = −0.003, and ΔSRMR = 0.001. Adding equality constraints on item thresholds also resulted in negligible changes, ΔCFI < 0.001, ΔRMSEA = −0.009, and ΔSRMR = 0.001.

A similar pattern was found in the primary school sample. The configural model fitted the data well, χ^2^(96) = 124.64, CFI = 0.962, RMSEA = 0.044, and SRMR = 0.054. Both loading invariance, ΔCFI = +0.003, ΔRMSEA = −0.004, and ΔSRMR = +0.007, and scalar invariance, ΔCFI = +0.001, ΔRMSEA = −0.002, and ΔSRMR = +0.001, were supported.

In the junior high school sample, the configural model showed acceptable fit, χ^2^(96) = 120.53, CFI = 0.950, RMSEA = 0.047, and SRMR = 0.062. Although absolute fit was somewhat weaker after additional constraints were imposed, changes in the approximate fit indices remained within the prespecified criteria for both loading invariance, ΔCFI = −0.005, ΔRMSEA = +0.001, and ΔSRMR = +0.015, and scalar invariance, ΔCFI = −0.005, ΔRMSEA < 0.001, and ΔSRMR = +0.003.

Taken together, the results supported configural, loading, and scalar invariance across gender within all three educational-stage groups. The evidence was particularly clear in the university and primary school samples, whereas the junior high school models showed comparatively weaker absolute fit but acceptable changes across successive invariance levels.

#### 3.3.4 Measurement invariance across educational-stage groups

Measurement invariance was then examined across university students (*n* = 592), primary school students (*n* = 305), and junior high school students (*n* = 233). The configural model showed good fit, χ^2^(144) = 373.06, *p* < 0.001, CFI = 0.973, RMSEA = 0.065, and SRMR = 0.038. The loading-invariance model also showed acceptable fit, χ^2^(160) = 388.30, *p* < 0.001, CFI = 0.973, RMSEA = 0.062, and SRMR = 0.039. Changes in fit were small, ΔCFI < 0.001, ΔRMSEA = −0.003, and ΔSRMR = 0.001, supporting loading invariance.

The threshold-invariance model retained acceptable absolute fit, χ^2^(224) = 591.68, *p* < 0.001, CFI = 0.956, RMSEA = 0.066, and SRMR = 0.043. However, the decrease in CFI was 0.017, although changes in RMSEA and SRMR remained small, ΔRMSEA = 0.004 and ΔSRMR = 0.004. Evidence for full threshold invariance was therefore mixed. Accordingly, the results support the comparability of the factor configuration and loadings across educational-stage groups, but do not provide sufficient evidence for direct comparisons of observed or latent factor means.

#### 3.3.5 General discussion

The present research evaluated the factor structure, internal consistency, and measurement invariance of the Chinese version of the Conceptions of the Nature of Athletic Ability Questionnaire-2 (CNAAQ-2-C). Across three studies, the findings provided converging evidence for four related first-order dimensions: Stable, Gift, Improvement, and Learning. Study 1 identified this structure using ordinal exploratory factor analysis, and Study 2 replicated it in an independently recruited university sample using confirmatory factor analysis. Study 3 further examined the four-factor model in university, primary school, and junior high school samples and evaluated its measurement invariance across gender and educational-stage groups. Overall, the structural evidence was strongest in the university samples. The four-factor model was also supported in the primary school sample, whereas model fit and internal consistency were less favourable in the junior high school sample. Configural, loading, and threshold invariance were supported across gender. Across educational-stage groups, configural and loading invariance were supported, whereas evidence for threshold invariance was mixed.

#### 3.3.6 The four-factor structure of the CNAAQ-2-C

The present findings support Stable, Gift, Improvement, and Learning as four related but distinguishable dimensions of athletic ability beliefs in Chinese university students. In Study 1, the ordinal EFA showed that the four-factor solution provided the best overall fit among the one- to four-factor solutions. This structure was replicated in the independent Study 2 sample, where the correlated four-factor model showed good fit and all items loaded moderately to strongly on their intended factors. The convergence of the exploratory and confirmatory findings strengthens the evidence for the four-dimensional structure (Knekta et al., 2019).

Previous research has differed in whether these four dimensions are best interpreted solely as first-order factors or as components of a higher-order structure. The original CNAAQ-2 specified Stable and Gift under Entity beliefs and Improvement and Learning under Incremental beliefs (Biddle et al., 2003), and this hierarchical structure was subsequently supported in British and Singaporean secondary-school samples (Wang et al., 2005). The Dutch adaptation, however, reported acceptable fit for both the four-factor model and a model incorporating the two higher-order factors (Weltevreden et al., 2022). These findings suggest that the four first-order dimensions have been relatively reproducible, whereas evidence for their organisation into two broader factors has been less uniform.

In the present study, the higher-order model fitted the data substantially less well than the correlated four-factor model. In particular, Improvement and Learning showed relatively modest loadings on the higher-order Incremental factor. The comparison should nevertheless be interpreted cautiously because each higher-order factor was represented by only two first-order indicators and required identification constraints. Within these limitations, the current data provide stronger support for interpreting Stable, Gift, Improvement, and Learning as four correlated first-order dimensions than for summarising them using two higher-order scores. The four subscales are therefore treated as the principal scoring and interpretive units of the CNAAQ-2-C, while Entity and Incremental remain useful as broader theoretical categories.

Item 4 required particular attention within the four-factor structure. In the EFA, it had a relatively modest loading on Gift and also cross-loaded on Learning, indicating less differentiated performance than the other items. In the independent CFA sample, however, Item 4 had a standardized loading of 0.736 on Gift. The 11-item model excluding Item 4 showed somewhat better descriptive fit and a lower Gift-Learning correlation, suggesting that the item contributed some overlap between the two dimensions. Nevertheless, the original 12-item model already showed good fit, and deleting Item 4 would leave Gift with only two indicators, narrow the content represented by the subscale, and would reduce comparability with the original instrument. Retaining Item 4 therefore reflects a balance among factor differentiation, content coverage, and continuity with the original CNAAQ-2, rather than a conclusion that the item performed without limitation. This decision is consistent with the view that item retention should consider both empirical performance and construct representation rather than model fit alone (Clark and Watson, 2019).

One tentative explanation for the EFA cross-loading is that respondents may not view natural aptitude and subsequent learning as mutually exclusive. This interpretation cannot be confirmed from the present data, because the translation process did not identify Item 4 as problematic and no cognitive interviews were conducted. Further item-level research should therefore examine whether the pattern reflects the breadth of the Chinese wording, sample-specific variation, or conceptual overlap between Gift and Learning.

#### 3.3.7 Measurement comparability across gender and educational stages

Gender and educational-stage analyses yielded different levels of measurement comparability. Separate analyses within the university, primary school, and junior high school samples supported configural, loading, and scalar invariance across gender. This suggests that the four-factor structure, item–factor relations, and item intercepts were broadly comparable between male and female participants within each educational-stage group. Although the junior high school models showed somewhat weaker absolute fit than the university and primary school models, changes in fit across successive invariance levels remained within the prespecified criteria. These findings provide a basis for examining gender differences in latent means and structural relations within each educational-stage group using appropriate multigroup models.

Across university, primary school, and junior high school students, configural and loading invariance were supported, indicating that the same four dimensions were represented and that the items related to their intended factors in broadly similar ways. Evidence for threshold invariance was less conclusive: changes in RMSEA (ΔRMSEA = 0.004) and SRMR (ΔSRMR = 0.004) were small, whereas the decrease in CFI (ΔCFI = −0.017) exceeded the prespecified criterion. Thus, the findings support examination of structural relations across educational-stage groups, but these relations should be compared through formal multigroup tests rather than through differences in within-group significance. By contrast, direct comparisons of observed or latent factor means are not supported. Accordingly, the means reported for the three groups are descriptive and should not be interpreted as substantive educational-stage differences (Putnick and Bornstein, 2016).

#### 3.3.8 Internal consistency and performance in younger samples

Internal consistency estimates were higher and more consistent in the university samples than in the younger groups. Learning in the primary school sample and Gift and Improvement in the junior high school sample yielded estimates close to or below 0.70, with the lowest α and ω values being approximately 0.65 for Improvement among junior high school students. Because each CNAAQ-2-C subscale contains only three items, its internal consistency estimates are partly constrained by scale length (Cortina, 1993). However, the similarly low α and ω estimates for some subscales suggest that the pattern was not specific to coefficient alpha alone (McNeish, 2018).

The group-specific CFA results showed a similar pattern. The four-factor model fitted the university and primary school samples well but showed weaker fit in the junior high school sample. Although the standardized loadings remained moderate to substantial, the combination of weaker model fit and lower reliability suggests less robust psychometric performance in this group.

Taken together, the weaker model fit and lower reliability estimates indicate less robust psychometric performance in the junior high school sample than in the university and primary school samples. The source of this variation cannot be determined from the present data. It may reflect the brevity of the subscales, differences in item comprehension or response-category use, administration conditions, or other sample-specific characteristics. Cognitive and linguistic demands can affect children’s responses to questionnaires (Borgers et al., 2000), but the present study did not include cognitive interviews and therefore could not directly assess how participants at different educational stages interpreted the items.

Accordingly, the CNAAQ-2-C may be used cautiously for group-level research in primary and junior high school samples, with subscale-specific reliability evaluated and reported in each new sample.

#### 3.3.9 Implications for measurement and use

The current findings support Stable, Gift, Improvement, and Learning as the primary scoring and interpretive units of the CNAAQ-2-C. Although Entity and Incremental remain useful theoretical categories, the corresponding higher-order model was less consistent with the present data. Use of broader Entity and Incremental scores would therefore require additional evidence for the higher-order structure. Retaining the four subscales also preserves distinctions between stability and innate talent, and between improvement through practice and learning.

The invariance results also define the limits of group comparisons. Among university students, gender differences in latent means and structural relations may be examined using appropriate multigroup models. Across educational stages, the common factor structure and comparable loadings support comparisons of structural relations, but the mixed evidence for threshold invariance does not support direct comparisons of observed or latent means. Raw-score differences across educational-stage groups should therefore not be interpreted as substantive group differences.

Further validation should focus on how respondents understand the items rather than only on additional model testing. Cognitive interviews across educational levels could clarify the interpretation of key concepts and response categories, particularly for Item 4 and the less reliable subscales in younger samples (Beatty and Willis, 2007). Such evidence would help determine whether further wording adaptation is needed before broader use in younger populations.

## 4 Limitations and future directions

Several limitations should be acknowledged. First, the samples were recruited from Guangdong Province and were not selected to represent the broader Chinese student population. Replication in samples from other regions and educational settings is needed to evaluate the generalizability of the present findings.

Second, although the adaptation involved independent forward translations, reconciliation, and back-translation, the present study did not include cognitive interviews or a formal content-validity evaluation with members of the target populations. The structural analyses therefore cannot establish whether students at different educational stages understood the items as intended or considered them relevant to their experiences (Terwee et al., 2018). This limitation is particularly relevant to Item 4, which showed some overlap with Learning in the exploratory sample. Cognitive interviews and further item-level analyses are needed to determine whether this pattern reflects the translated wording, sample-specific variation, or conceptual overlap between natural aptitude and learning.

Third, the educational-stage groups were recruited from different settings and may also have differed in administration conditions, sport experience, and other unmeasured characteristics. The cross-sectional design does not allow the observed variation in model fit and reliability to be attributed specifically to educational stage. Future studies should use more closely matched samples and standardized administration procedures when evaluating measurement properties across educational levels.

Fourth, all four subscales contained only three items, and some internal consistency estimates were below 0.70 in the younger samples. These findings indicate limited score precision for some subscales, particularly in junior high school students. Further research should first examine item comprehension and item functioning before considering whether wording revisions or additional indicators are needed.

Finally, the present studies focused on factor structure, internal consistency, and measurement invariance. Test–retest reliability and associations with theoretically relevant external constructs were not examined in the revised analyses. Future validation studies should evaluate these forms of reliability and validity evidence using prespecified hypotheses and measures appropriate to each target population.

## 5 Conclusion

The present research provides initial evidence for a four-factor Chinese version of the CNAAQ-2 comprising Stable, Gift, Improvement, and Learning beliefs. The structure was identified in an ordinal EFA and replicated in an independent university sample. Configural, loading, and threshold invariance were supported across gender among university students, whereas configural and loading invariance were supported across educational-stage groups. Evidence was less consistent for threshold invariance and for the reliability of some subscales in younger samples. The CNAAQ-2-C is therefore most strongly supported for group-level research with university students, with more cautious interpretation warranted in primary and junior high school samples. Further content-validity work, item-level evaluation, and replication are needed before educational-stage mean comparisons or individual-level applications can be recommended.

## Data Availability

The raw data supporting the conclusions of this article will be made available by the authors, without undue reservation.
