# Supplementary material for: Psychometric evaluation of the Chinese CNAAQ-2: factor structure and measurement invariance across gender and educational stages
Source: Front Psychol. 2026 Sep 9;17:1882376. doi: 10.3389/fpsyg.2026.1882376 (PMC13597683; doi:10.3389/fpsyg.2026.1882376)
Supplement: Supplementary file 1 [file Table_1.docx]

| **Table S1**  *English and Chinese Versions of the CNAAQ-2-C Items* | |
| --- | --- |
| **Item** | **题目** |
| **Stable** | **稳定** |
| 1. You have a certain level of ability in sport and you cannot really do much to change that level. | 1. 你的体育运动水平是有限度的，要提高是很难的。 |
| 3. Even if you try, the level you reach in sport will change very little. | 3. 即使你很努力，你的运动技术水平也只能提升一点点。 |
| 10. It is difficult to change how good you are at sport. | 10. 要进一步提升你的体育运动能力是一件很困难的事情。 |
| **Gift** | **天赋** |
| 4. You need to have certain ‘gifts’ to be good at sport. | 4. 你需要有一定的“天分”才能在体育运动领域取得好成绩。 |
| 7. To be good at sport, you need to be born with the basic qualities which allow you success. | 7. 要想在体育领域取得好成绩， 你需要有与生俱来的身体素质。 |
| 11. To be good at sport you need to be naturally gifted. | 11. 要在体育领域取得一定的成绩，你需要与生俱来的天赋。 |
| **Improvement** | **增进** |
| 6. In sport, if you work hard at it, you will always get better. | 6. 在体育领域，只要你刻苦和努力，你就一定取得进步。 |
| 9. How good you are at sport will always improve if you work at it. | 9. 不管你的运动水平怎样，只要你付出，你就一定会取得进步。 |
| 12. If you put enough effort into it, you will always get better at sport. | 12. 如果你付出足够的努力，你一定会在体育领域取得更好的成绩。 |
| **Learning** | **学习** |
| 2. To be successful in sport you need to learn techniques and skills, and practise them regularly. | 2. 要在体育运动领域获得成功，你需要学习各种技术和技能，并经常练习。 |
| 5. You need to learn and to work hard to be good at sport. | 5. 你需要学习和努力练习才能在体育领域取得好成绩。 |
| 8. To reach a high level of performance in sport, you must go through periods of learning and training. | 8. 要想在体育领域达到一定的高水平表现， 你必须经历一定时期的学习和训练。 |
